# Epigenetic Modulation as a Therapeutic Prospect for Treatment of Autoimmune Rheumatic Diseases

**DOI:** 10.1155/2016/9607946

**Published:** 2016-08-10

**Authors:** Marzena Ciechomska, Steven O'Reilly

**Affiliations:** ^1^National Institute of Geriatrics Rheumatology and Rehabilitation, Department of Pathophysiology and Immunology, Warsaw, Poland; ^2^Faculty of Health and Life Sciences, Northumbria University, Newcastle upon Tyne, UK

## Abstract

Systemic inflammatory rheumatic diseases are considered as autoimmune diseases, meaning that the balance between recognition of pathogens and avoidance of self-attack is impaired and the immune system attacks and destroys its own healthy tissue. Treatment with conventional Disease Modifying Antirheumatic Drugs (DMARDs) and/or Nonsteroidal Anti-Inflammatory Drugs (NSAIDs) is often associated with various adverse reactions due to unspecific and toxic properties of those drugs. Although biologic drugs have largely improved the outcome in many patients, such drugs still pose significant problems and fail to provide a solution to all patients. Therefore, development of more effective treatments and improvements in early diagnosis of rheumatic diseases are badly needed in order to increase patient's functioning and quality of life. The reversible nature of epigenetic mechanisms offers a new class of drugs that modulate the immune system and inflammation. In fact, epigenetic drugs are already in use in some types of cancer or cardiovascular diseases. Therefore, epigenetic-based therapeutics that control autoimmunity and chronic inflammatory process have broad implications for the pathogenesis, diagnosis, and management of rheumatic diseases. This review summarises the latest information about potential therapeutic application of epigenetic modification in targeting immune abnormalities and inflammation of rheumatic diseases.

## 1. Autoimmune Rheumatic Diseases

Systemic autoimmune rheumatic diseases are characterised by pain and chronic joint inflammation. There are more than 200 different conditions that are labelled as rheumatic diseases including rheumatic arthritis, systemic sclerosis, systemic lupus erythematosus, psoriatic arthritis, ankylosing spondylitis, and Sjogren syndrome. Moreover, autoimmune rheumatic diseases share many common features, which makes them difficult to differentiate within the group. Indeed, up to 50 percent of patients with autoimmune rheumatic diseases cannot be easily categorised with a specific disorder in the first 12 months of follow-up [1].

One of the major characteristics of rheumatic diseases is chronic inflammation and autoimmunity, which consequently leads to tissue destruction and reduces patients' mobility. Immune cells play a key role in inflammation due to involvement in initiation and maintenance of the chronic inflammatory stages. In particular, circulating monocytes that may differentiate towards macrophages or dendritic cells are able to produce proinflammatory cytokines including interleukin-1 (IL-1), IL-6, IL-8, and tumour necrosis factor-*α* (TNF-*α*) [2]. Monocytes are also responsible for the production of inflammatory mediators including reactive oxygen species (ROS) and cyclooxygenase-2 (COX-2) [3]. COX-2 is a key enzyme in prostaglandins biosynthesis driving the inflammatory response. Monocytes can produce chemokines which attract T and B cells for the secretion of proinflammatory cytokines. Activated B cells are able to present autoantigens and produce autoantibodies maintaining the inflammatory process leading to tissue destruction. The presence of autoantibodies is a hallmark of autoimmune rheumatic diseases [4]. Also, a subset of helper CD4+ T cells, Th17, is reported to be involved in rheumatic pathogenesis [5]. Th17 cells are characterised by production of IL-17. This interleukin is a potent proinflammatory cytokine that amplifies ongoing inflammation by induction of TNF-*α*, IL-1*β*, and IL-6 in macrophages as well as of other cell types such as keratinocytes, fibroblasts, and synoviocytes. Fibroblast-like synoviocytes (FLS) located inside joints in the synovium also play a key role in pathogenesis of rheumatic diseases due to their production of proinflammatory cytokines, adhesion molecules, and matrix proteases contributing to cartilage destruction. Rheumatoid FLS develop a unique autoaggressive phenotype that increases invasiveness into the extracellular matrix, promotes inflammatory cell recruitment, and elevates production of COX-2. NSAIDs are widely used anti-inflammatory agents that act through the inhibition of the COX enzymes. Although COX-inhibitors lead to reduced synthesis of prostaglandins at the site of inflammation, suppression of gastrointestinal or renal prostaglandins synthesis is associated with mechanism-based toxicities. This limits the usefulness of these otherwise potent drugs. In addition, COX-2 inhibitors have been found to increase the risk of myocardial infarction. Thus, finding new agents which will specifically block inflammation may provide therapeutic opportunities in immune-mediated rheumatic diseases.

## 2. Overview on Epigenome-Influencing Drugs

Epigenetics is defined as reversible and heritable changes in gene function without alteration of the underlying DNA sequence itself [6]. Epigenetic mechanisms are sensitive to external stimuli, bridging the gap between environmental and genetic factors. In particular, monozygotic (MZ) twins do not show complete concordance for many complex diseases. MZ discordance rates for autoimmune diseases are 20–80 percent, indicating a substantial role of epigenetic factors in the development of these disorders [7, 8]. Indeed, it has been reported that epigenetic mechanisms mediate development of chronic inflammation by modulating the expression of proinflammatory cytokines including TNF-*α*, IL-6, and IL-1 and induction of COX-2 and transcription factor NF-*κ*B. These molecules are constitutively produced by a variety of immune cells under chronic inflammatory conditions, which consequently leads to the development of many diseases including cancer, cardiovascular diseases, or autoimmune rheumatic disorders.

## 3. Noncoding RNA

Three main epigenetic mechanisms have been described including noncoding RNA species, DNA methylation, and histone modification. The first group of noncoding RNAs includes microRNA (miRNA) and long noncoding RNA (lncRNA). MicroRNAs (miRNAs) are endogenous, single-stranded RNAs of 19–25 nucleotides in length which can negatively regulate gene expression on posttranscriptional level. In particular, miRNA can hybridise to 3–8 nucleotides within 3′-untranslated region (3′UTR) of target messenger RNA (mRNA) referred to as “seed sequence” [9]. The formation of such miRNA-mRNA duplexes leads to mRNA degradation or translational repression. miRNAs have been studied extensively due to their role in regulation of almost every cellular process. It is known that miRNAs can act as a fine-tuner of gene expression and can negatively regulate approximately 30 percent of human protein-coding genes [10]. In addition, miRNAs are attractive as potential biomarkers. Some of miRNAs have been already tested in preclinical studies that aimed to treat cancer including lung, prostate, or leukemia [11]. Interestingly, randomised, phase IIa, double blind clinical trial (test number NCT01200420) has been conducted to treat hepatitis C virus (HCV) using locked nucleic acid inhibitor of miRNA-122 (Table 1). miRNA-122 is crucial for viral replication in hepatocytes; thus the reintroduction of miRNA-122 inhibitor significantly reduced virus replication [12, 13]. Recent phase I clinical trial has also tested the drug called MRX34. MRX34 is a double-stranded miRNA-34 encapsulated in liposomal nanoparticles. miRNA-34a represses the expression of more than 20 oncogenes which results in inhibition cancer cell viability, stemness, metastasis, or chemoresistance. Thus, MRX34 is widely tested in solid tumours and hematological malignancies [9]. Many studies have also shown the role of lncRNA in diverse cellular processes. lncRNAs are non-protein-coding transcripts longer than 200 nucleotides regulating gene expression. However, the exact functional roles and mechanisms of lncRNAs are still unclear.

## 4. DNA Methylation

Another mechanism of epigenetic changes is DNA methylation induced by a highly conserved family of DNA methyltransferases (DNMTs). DNMT1 is the most abundant DNA methyltransferase in mammalian cells [14]. The insertion of methyl group to cytosine at the carbon 5 position leads to structural changes in chromatin and is mostly associated with gene silencing. In humans, methylation mainly occurs when cytosine is followed by guanine and is linked with phosphate called CpG islands. Approximately 1 percent of the genome consists of CpG islands [11]. Also, it is reported that roughly 60–70 percent of human genes are linked to promoter CpG islands which suggests that methylation of CpG island is an important regulatory mechanism of gene expression [12]. It has been shown that vitamin B12 rich diet (B vitamins acted as methyl donors) in agouti mouse model prevented from development of inflammation mediated diabetes and cancer. In contrast, mice which did not receive vitamin B were predisposed for these diseases [14]. Therefore, methylation plays a key role in physiological conditions and the alteration in DNA methylation signature can have impact on disease development. One of the Food and Drug Administration- (FDA-) approved drugs inhibiting DNA methylation is 5′-azacytidine (commercial name Vidaza). This drug has been already used in phase III randomised, controlled trial to treat myelodysplastic syndrome and leukemia [15]. Similar inhibitory effect on DNA methyltransferases has the 5-aza-2′-deoxycytidine or 5′-AZA (known under commercial name Decitabine), which is used to treat many types of cancer and the myelodysplastic syndrome [16, 17]. DNA hydroxymethylation is also epigenetic modification mediated by Ten-Eleven Translocation (TET) family proteins which were discovered relatively recently [18]. TET enzymes are dioxygenases capable of oxidizing the methyl group of 5-methylcytosine (5mC) and converting 5mC into 5-hydroxymethylcytosine (5hmC), which results in DNA demethylation. It has been shown that the increased expression of TET1–TET3 enzymes in monocytes and TET2 in T cells leads to aberrant global DNA hydroxymethylation of early RA patients [19]. Interestingly, treatment with methotrexate partially reduces the DNA hydroxymethylation level. Indirect TET inhibition induced by AGI-5198 compound leads to growth suppression and promotes differentiation of glioma cells [20]. Similarly, HMS-101 inhibitor limits the growth of acute myeloid leukemia cells suggesting potential therapeutic application of TET inhibitors in cancer and also in rheumatic diseases [21].

## 5. Histone Modification

**Figure 1:**
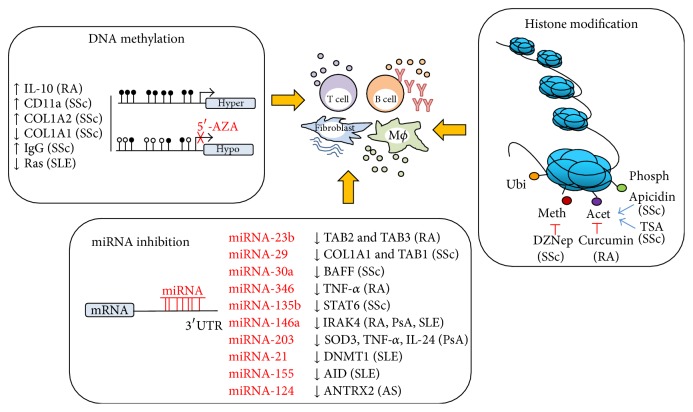
Epigenetic agents modulating immune response in rheumatic diseases including RA, SSc, SLE, AS, and PsA. Schematic of the epigenetic modulations represented by DNA methylation, histone modification, and RNA interference influencing immune cells (B cells, T cells, and monocytes) and fibroblasts.* DNA methylation* refers to covalent addition of a methyl group to the 5-position of the cytosine ring, which can be inhibited by 5′-AZA. 5′-AZA induces DNA hypomethylation and drives differential gene expression.* Histone modifications* are reversible and site-specific histone alterations including acetylation (Acet), methylation (Meth), phosphorylation (Phosph), or ubiquitination (Ubi). Histone methylation or acetylation can be either activated by apicidin and TSA or inhibited by DZNep and curcumin.* miRNA inhibition* is a formation of miRNA-mRNA duplexes in the position of 3′UTR. This leads gene silencing (genes in black) by specific miRNAs (in red).

Another epigenetic phenomenon is histone modification. This modification alters the electrostatic charge of the histones resulting in conformational changes in protein binding sites and facilitating or blocking DNA accessibility. Histone modifications can be mostly represented by acetylation, methylation, phosphorylation, ubiquitination, ribosylation, citrullination, biotinylation, and sumoylation of histone N-terminal tail domains and also core domains [22]. It is believed that the histone acetylation is usually associated with increased binding of transcription factors to nucleosomal DNA and facilitates transcription initiation, whereas histone methylation can either activate or repress gene expression. Acetylation removes the positive charge on the histones and reduces the interaction between histones and negatively charged phosphate groups on DNA [23]. Therefore, the condensed heterochromatin is transformed into a more relaxed euchromatin that is associated with greater levels of gene transcription. Vorinostat (also known as suberanilohydroxamic acid, SAHA) has been shown to bind to the active site of histone deacetylases (HDACs). HDACs catalyse the removal of acetyl group from lysine residue. Inhibition of HDACs by Vorinostat leads to accumulation of hyperacetylated histones. This drug was the first histone deacetylase inhibitor to be approved by FDA (2006) to treat cutaneous T cell lymphoma with substantial response rates over 30 percent in patients [24]. Unlike in the cancer field, there is still no epigenetics-based drug on the market to treat rheumatic disorders. Finding new agents is greatly needed, because the economic burden of rheumatic diseases is substantial. Their cost is estimated at more than 200 billion Euros per year in Europe and they are the most expensive of all diseases for the European health care systems [25]. This review highlights the impact of chronic inflammation and immune disability on globally disturbed DNA methylation pattern, aberrant histone modification' profile, and divergent miRNA signature that is observed in autoimmune rheumatic diseases. However, it is a chicken or egg dilemma and it needs to be further investigated to find out whether inflammation triggers epigenetic changes or epigenetic alteration drives inflammation. Epigenome-influencing drugs may have future impact on diagnosis and/or therapeutics of rheumatic diseases. Epigenetic mechanisms, which modify immune cells and fibroblasts in rheumatic diseases, are depicted in Figure 1.

## 6. Rheumatoid Arthritis

Rheumatoid arthritis (RA) is the most common inflammatory joint disease which affects approximately 1% of the population worldwide with unknown etiology. RA is a chronic autoimmune inflammatory condition which is characterised by an influx of inflammatory cells from the blood stream into the synovial membrane or synovial fluid. Such influx of immune cells producing inflammatory cytokines results in progressive erosion of articular cartilage. Phagocytes, B cells, and T cells are the most prominent cells in the rheumatoid synovium. Macrophages along with granulocytes are an important source of proinflammatory cytokines, chemokines, and reactive oxygen species (ROS) that accompany inflammatory processes [4]. On the other hand, antigen-specific B cells are involved in autoantigens presentation to T cells and in production of autoantibodies, which mediates in joint destruction. In addition, the presence of ectopic follicular structures in chronically inflamed tissues resembling germinal centres provides strong evidence of ongoing immune reactions [26]. Recent studies have indicated that miRNA plays a critical role in pathogenesis of RA. Raj and Mufti showed that miRNA-346 regulates TNF-*α* synthesis (one of the major proinflammatory cytokines involved in the pathogenesis of RA) in LPS stimulated synovial fibroblasts [15]. The level of miRNA-146a is significantly upregulated in CD4+ T cell subset and positively correlates with TNF-*α* concentration in RA patients [16, 17]. Also, the level of miRNA-150 is elevated in IL-17 producing T cells [27]. In contrast, Zhang et al. reported that miRNA-23b inhibits IL-17-associated autoimmune inflammation by targeting TGF-*β* binding protein 2 (TAB2) and TAB3 [18]. Emerging evidence revealed that lncRNAs have various expression in autoimmune diseases. It has been shown that adalimumab (anti-TNF-*α* antibody) and tocilizumab (anti-IL-6R antibody) treated RA patients have differential expression of 85 lncRNAs in CD14+ monocytes [28]. Similarly, Song et al. have shown elevated expression level of lncRNAs called Hotair in PBMC and serum exosome, suggesting that lncRNAs could be used as potential biomarkers for diagnosing RA [29], while overexpression of Hotair by introduction of lentiviral construct results in decreased expression of MMP-2 and MMP-13 in FLS from RA patients. DNA methylation pattern is also impaired in RA affecting immune-related genes and consequently influencing immune responses. In particular, global DNA hypomethylation is observed in peripheral blood mononuclear cells (PBMCs) derived from RA patients [30]. It has been shown that hypomethylated promoter region of chemokine CXCL12 leads to increased MMPs expression and joint destruction in RA patients [31]. Similarly, the methylation levels of IL-6 promoter in PBMCs was significantly lower in RA patients than those in controls [32]. In contrast, gene coding for dual specific phosphate 22 (DUSP22) is hypermethylated in T cells of RA and in Sjogren's syndrome patients [33, 34]. DUSP22 is a tyrosine phosphatase which negatively regulates the IL-6 transcription factor STAT3. It is known that IL-6 plays a pivotal role in chronic inflammation in autoimmune diseases [35]. Another group has identified that the promoter of anti-inflammatory cytokine IL-10 is hypermethylated in four different regions of CpG site [36]. The level of IL-10 which is mainly produced by monocytes and T reg cells is reduced in RA. It has been shown that PBMCs treated with 5′-AZA have increased production of IL-10. This suggests that specific demethylation of IL-10 promoter induced by 5′-AZA can prevent development of RA by induction of anti-inflammatory IL-10 and suppression of immune responses. It has been also found that alteration in histone modification can contribute to RA development. The balance of histone acetylase (HAT) versus HDAC is strongly shifted towards chronic histone hyperacetylation in RA patients [37]. This consequently leads to proinflammatory genes expression including IL-6 and IL-8. Indeed, the level of histone H3 acetylation in the IL-6 promoter is significantly elevated in RA synoviocytes resulting in enhanced IL-6 secretion and joint destruction [38]. Surprisingly, treatment with curcumin abrogated H3 acetylation and reduced IL-6 secretion which suggests that epigenetic mechanisms are implicated in targeting RA pathogenesis.

## 7. Systemic Sclerosis

Systemic sclerosis (SSc) is an autoimmune connective tissue disease characterised by autoimmunity, vascular abnormalities, and fibrosis via accumulation of extracellular matrix (ECM) proteins. Uncontrolled fibrosis progression often results in dysfunction of the affected organs and consequently leads to premature death in SSc patients [39]. The role of miRNA has also been demonstrated in SSc pathogenesis. It has been shown that the family of miRNA-29 plays a pivotal role in SSc skin fibrosis by targeting collagen expression [19, 20]. In addition, we have shown that SSc fibroblasts are able to reverse fibrotic phenotype following miRNA-29 transfection. We found that miRNA-29 can modulate its novel target gene, TAB1, and that it downregulates tissue inhibitor of metalloproteinases-1 (TIMP-1) expression as a result of TAB1 degradation [21]. Similarly, transfection of miRNA-30a-3p in IFN-*γ*-activated SSc fibroblasts decreases synthesis of B cell-activating factor (BAFF) [40]. BAFF plays a central role in the survival and homeostasis of B cells and plasma cells. Autoreactive B cells are strongly dependent on BAFF presence and the increased level of BAFF correlates with high autoantibody titers and with disease activity in SSc. Therefore, miRNA-30 targeting BAFF expression could be used in SSc therapy. In addition, 5′-AZA-treated SSc T cell has shown increased CD11a expression, whereas 5′-AZA-treated SSc T cells cocultured with either B cells or fibroblasts resulted in increased production of IgG or COL1A2, respectively [41]. These data suggest that demethylation of CD11a regulatory elements and subsequent CD11a overexpression in T cells may mediate immunological abnormalities and fibrotic processes in SSc. Therapies which reduce CD11a due to specific DNA methylation are needed in SSc. On the contrary, our latest results have shown that 5′-AZA-treated fibroblasts decreased expression of collagen and upregulated the miRNA-135b expression level. miRNA-135b targets STAT6 and attenuates the IL-13-induced collagen expression. This indicates that specific targeting DNA methylation may represent a novel therapeutic approach for the treatment of SSc.

Another hallmark of SSc is perivascular infiltration of immune cells, mainly monocytes, which are the first immune cells to infiltrate the SSc skin. The results from our group demonstrated that circulating monocytes from SSc patients contribute to the imbalance between TIMP-1 and MMPs and to increased profibrotic IL-6 production upon TLR8 agonist stimulation (ssRNA) [42–44]. Interestingly, we have also shown that epigenetic modification induced by DZNep (histone methyltransferases) or apicidin (inhibitor of histone acetylases) in SSc monocytes can modulate TIMP-1 expression and subsequently fibroblasts transdifferentiation [44]. Another study has shown that global H4 but not H3 acetylation of SSc B cells was positively correlated with disease activity and that the expression of HDAC2 protein was negatively correlated with skin thickness [45]. This clearly indicates that epigenetic alteration plays an important role in the pathogenesis of SSc.

## 8. Psoriatic Arthritis

Psoriatic Arthritis (PsA) is a chronic inflammatory skin disease with unknown etiology. The interactions between genetics and the environmental factors in PsA are still not well defined. The disease is characterised by abnormal proliferation and differentiation of keratinocytes. In addition, infiltration of immune cells which secrete high level of various immune-regulated inflammatory cytokines and chemokines is observed in PsA. Recently, imbalance in epigenetic networks has been indicated to be an important element in psoriasis development. Several studies have shown that differentially expressed miRNAs levels play a role in psoriasis pathogenesis. In particular, it has been reported that miRNA-203 expression is downregulated in psoriatic lesion. Based on bioinformatic analysis, miRNA-203 targets gene suppressors of cytokine signalling 3 (SOC3). SOC3 is involved in negative regulation of the IL-6 transcription factor STAT3. Furthermore, miRNA-203 directly targets TNF-*α* and proinflammatory IL-24 in primary keratinocytes [46]. Another group identified that miRNA-146a is also dysregulated in psoriatic lesions. miRNA-146a targets the TNF receptor-associated factor 6 (TRAF6) and the IL-1 receptor-associated kinase 1 (IRAK1). Activation of IRAK1 triggers the production of TNF-*α*, IL-6, IL-8, and IL-1*β*. Xia et al. also found that the increased level of miRNA-146a is positively correlated with the Psoriasis Symptom Inventory (PSI) score [47]. In contrast, the addition of anti-TNF-*α* blocking antibody reduced the level of miRNA-146a in patients' serum. These data suggest that overexpression of miRNA-203 and miRNA-146a may be useful in repression of the immune-mediated inflammation process and may provide potential therapeutic strategy in psoriasis pathogenesis. Another study reported that the DNA methylation pattern is changed in psoriatic skin in comparison to normal tissue. They showed strong correlation between S100 Calcium Binding Protein A9 (S100A9) and DNA methylation signature of psoriasis patient samples fallowing phototherapy [48]. S100A9 is a calcium binding protein which plays a prominent role in regulation of inflammatory processes and immune response. Also, Gervin et al. have demonstrated that monozygotic twins (MT) have a different methylation pattern between an unaffected twin and a twin suffering for PsA [8]. They showed the differences in DNA methylation pattern of proinflammatory TNF-*α* ligand 11 also known as the receptor activator of nuclear factor kappa-B ligand (RANKL) in MZ twins. Moreover, DNA methylation signature of the arachidonate 5-lipoxygenase-activating protein (ALOX5AP) gene is altered in psoriatic MZ twin. ALOX5AP is involved in catalysis of arachidonic acid regulating inflammation via leukotrienes production. Another study has shown that 50% of CpG islands in the promoter region of p16 gene are hypermethylated in psoriatic epidermis and correlated with diseases activity [49]. p16 is an antiapoptotic protein that supports the concept of an abnormal mechanism of hyperproliferative skin diseases. Abnormal expression of HATs and HDACs regulating gene expression has been also observed in PsA. Indeed, Ham et al. have shown that the promoter region of HDAC-6 is hypermethylated in naive CD4+ T cells in patients. Furthermore, the level of HDAC-1 in skin samples and PBMCs from PsA patients is increased compared to healthy subjects [50, 51]. These findings implicate that novel therapy for PsA should be also supplemented with agents altering the abnormal histone modification pattern.

## 9. Systemic Lupus Erythematosus

The etiology of Systemic Lupus Erythematosus (SLE) remains to be elucidated; however it is an autoimmune disorder with clear links to the innate and adaptive immune systems. Environmental triggers may initiate the disease on a genetic susceptibility background. SLE is a multiorgan disorder in which there are autoantibodies to DNA that are not only diagnostic of the disease but also pivotal in disease pathogenesis.

It was as early as 1990 that methylation abnormalities were first described in SLE T cells. The most convincing evidence comes from the fact that procainamide and 5′-AZA (both hypomethylating drugs) treated CD4+ T cells cause a lupus-like disease in mice [52]. This suggests a critical role of hypomethylation in T cells in mediating SLE [52]. It was suggested that decreased Ras signalling is involved also in DNA hypomethylation in T cells [53]. Other studies have suggested that growth arrest and DNA damage-induced gene 45*α* (GADD45*α*) are associated with DNA hypomethylation in SLE [54]. In further support of this it was demonstrated that increased oxidative stress in T cell in SLE may alter the expression of various proteins but also force downregulation of DNMT1 expression and thus hypomethylation [55]. Indeed, adoptive transfer of T cells modified by oxidative stress into syngeneic mice resulted in lupus-like disease with reduced methylation [56]. A study found that isolated T cells from SLE patients were globally hypomethylated. The genes methylated include CD11a and CD70 [57]. CD11a of course forms lymphocyte function-associated antigen 1 and would be important in immune responses. The X chromosome in women with lupus is hypomethylated suggesting a reason for the preponderance in females [58]. A large scale genomewide DNA methylation study in isolated CD4+ T cells from lupus patients found 236 hypomethylated CG sites. Enrichment of genes associated with apoptosis was found. A further study of DNA methylation in isolated T cells found that a large amount of interferon regulated genes is methylated differently. The authors suggest that there is an epigenetic alteration of interferon genes which explains the interferon response in SLE [59]. Similarly, it has been found that interferon regulated genes in CD4+ T cells of SLE patients that had quiescent disease are hypomethylated, which suggests that they are poised “to trigger” [60]. Recently, a study of SLE patients has demonstrated that altered DNA methylation pattern of interferon genes is associated with production of autoantibodies characterising SLE [61]. As well as T cells playing a critical role, B cells have also been described to be involved in SLE pathogenesis. Indeed, hypomethylation of SLE B cells has been described and blocking IL-6 with a monoclonal antibody restores B cell methylation levels. These data suggest that IL-6 is driving B cell alterations [62]. Interestingly, miRNA-155 targets Activation Induced Cytidine Deaminase (AICDA) which is critical in B cell development. It has been demonstrated that AICDA is dysregulated in SLE. miRNA-29b appears to be overexpressed in SLE CD4+ T cells and indirectly regulates hypomethylation by targeting DNMT1. DMNT1 is an enzyme which is important in DNA methylation [63]. The important negative regulators of TLR signalling are miRNA-146a and miRNA-29 which are dysregulated in SLE [64, 65]. It was also further shown that miRNA-21 additionally targets DNMT1 in SLE CD4+ T cells [66]. Interestingly, the authors demonstrated that targets of miRNA-146 included the interferon regulatory factor 5 and STAT1, a downstream target of IFN activation, and these are indeed dysregulated in SLE. A great study demonstrated that miRNA-3148 affects the stability and regulation of TLR7 [67]. TLR7 is the receptor for RNA and this is clearly important in SLE and is highly expressed on dendritic cells and may link RNA, TLR, and miRNAs together. In T cells miRNA-31 has also been found to be dysregulated in T cells and this dysregulation is associated with the impaired production of IL-2, a critical T cell growth factor [68]. It is clear that a multitude of miRNAs are dysregulated in SLE; therefore cell-free miRNAs have recently emerged as noninvasive biomarkers [69]. miRNA-146 and miRNA-155 have been found to be a possible biomarker in SLE derived from urinary sediment [69]. Abnormal histone modifications have also been described in SLE. It has been reported that in SLE patients there is a global histone hypoacetylation due to downregulation of Ezh2 enzyme. Ezh2 is involved in histone methylation [70]. The epigenetic modifying enzyme Ezh2 has been also found to be downregulated in the SLE T cells and this is one of the enzymes that methylate the histones [70]. Ezh2 has been also shown to be regulating the expression of the transcription factor STAT5 to epigenetically repress the immunoglobulin K chain complex, critical in B cell lineages [71]. Using the lupus-prone mouse model, it was found in the isolated T cells from this model that the HDACs were dysregulated suggestive of the mechanism of altered histone methylation [72]. Histone H3 trimethylation has also been described to be altered in SLE [73]. The major histone modifications which are implicated in SLE include methylation and acetylation and both are reversible. It has been shown in the lupus mouse model that introduction of TSA (a broad spectrum HDAC inhibitor) reduced IL-6 level and proteinuria [74]. CD70 is also elevated on CD4+ T cells from SLE patients and associated with higher dimethylated H3 lysine 4 in these patients [75].

## 10. Sjogren's Syndrome 

Sjogren's syndrome (SS) is an autoimmune disorder that affects the lacrimal and salivary glands, causing hypofunction which leads to dry eyes and dry mouth (xerostomia). There are a large prominent lymphocytic infiltrate in the salivary glands and also specific autoantibodies in the disease too. Patients with SS have a 20–40-fold increased risk of developing lymphoma. There are both associations with the innate and adaptive immune systems. Because the innate immune system has been heavily implicated in disease pathogenesis many studies have focussed on this system. One of the first miRNAs that has been shown to be dysregulated is miRNA-146a [76]. miRNA-146a has been found to be elevated in PBMCs of SS patients; however, the precise cell type of the PBMCs has not been confirmed [76, 77]. More importantly, Pauley et al. have shown that enhanced miRNA-16a levels in monocytes lead to increased phagocytosis in functional assays. This could represent a mechanism to help restore the altered phagocytosis seen in the disease. A follow-up study confirmed the increased miRNA-146a levels and the decreased target gene IRAK1 levels in PBMCs [78]. Using the minor salivary gland and whole miRNA arrays, a number of differentially regulated miRNAs in salivary glands from SS patients was found; however, their targets and the functional consequences of the differentially expressed miRNAs are still unknown [79]. A very interesting recent study showed that the SS related antigen B promotes global miRNA processing [80].

It has been recently shown that salivary gland epithelial cells are globally hypomethylated compared to controls [81]. A very interesting observation was that the global gland epithelial cells hypomethylation may be attributed to B cells as treatment with the B cell depleting antibody rituximab had more methylation. In isolated T cells from SS patients it has been found that they have lower FoxP3 expression levels, both mRNA and protein levels, and that this is associated with hypermethylation of the promoter region [82]. This observation could explain the reduced number of T reg cells in SS. A recent genomewide methylation study in isolated CD4+ T helper cells identified a multitude of genes that are differentially regulated in SS. The interferon pathways genes STAT1, IFI44L, and USP18 are all hypomethylated [83].

## 11. Ankylosing Spondylitis

Ankylosing spondylitis (AS) is a chronic and common inflammatory rheumatic disease characterised by new bone formation, ankyloses, and inflammation of the hips and spine. miRNA-16 and miRNA-221 are aberrantly expressed in T cells [84]. A functional SNP variant in miRNA-196a was found to be associated with Behcet's disease but not AS [85]. A recent study in AS demonstrated that miRNA-124 is elevated in peripheral blood cell of AS patients. miRNA-124 targets Anthrax Toxin Receptor 2 (ANTXR2) which is associated with risk of AS development [86]. Interestingly, inhibition of ANTXR2 by miR-mimics* in vitro* caused autophagy and subsequently protects T cells from apoptosis conferring advantage. Niu et al. have looked at common polymorphism in miRNA-146a associated with AS. However, no polymorphism was associated [87]. In AS, T cells are proposed to play a role; however, their precise role is unclear. It was shown that FoxP3 positive T cells are elevated in the inflamed joint in AS and that the FoxP3 locus is demethylated. This suggests that epigenetic mechanisms control FoxP3 expression [88]. Recently, in serum it was found that there were higher levels of SOCS1 methylation as compared to healthy controls and higher levels of SOCS methylation associated with higher IL-6 levels [89]. SOCS1 is the negative regulator of STAT1 signalling which is initiated after IL-6 stimulation; thus a reduction in the negative regulator of STAT1 would lead to unperturbed STAT1 signalling.

Reduced HDAC and HAT activities have been described in AS in PBMCs compared to controls [90]. The functional relevance of this is unknown because the balance between these two enzymes was not different. Furthermore, after anti-TNF-*α* therapy HAT activity increased in AS patients, with a clear increase in the HAT/HDAC balance [91]. Only one manuscript has demonstrated the altered histone methylation in CD4+ T cells in AS. In particular, it has been shown that specific AS SNP genotype has an altered histone modification profile and possibly alters binding to important transcription factors critical in the disease [92].

## 12. Giant Cell Arteritis

Giant Cell Arteritis (GCA) is a systemic autoimmune disease primarily affecting the elderly. It is characterised by inflammation of the large- and medium-sized arteries. GCA typically affects the temporal arteries. One of the most devastating features of the disease can be acute visual loss and patients can be present with ischaemic complications of the disease. One study found 853 hypomethylated genes in temporal arteries from GCA patients compared to controls. Many of these hypomethylated genes were associated with both Th1 and Th17 cells [93]. DNA methylation was also found to be altered in nuclear factor of activated T cells (NFAT), which was confirmed to be altered by immunohistochemistry [93]. NFAT is a critical factor mediating production of proinflammatory cytokines including IL-23. Thus, methylation regulation of NFAT may be crucial in driving the activation of Th17 cells in GCA. Only one report of dysregulated miRNA has been published in GCA and this study found that miRNA-21 was dysregulated in GCA temporal biopsies [94]. miRNA-21 was overexpressed in the biopsies and this appears to be tissue specific, as the use of PBMCs derived from the same donors when compared across groups demonstrated no difference. These data suggest that the increase of miRNA-21 level is tissue specific [94]. To this day only, a handful of studies have looked at the epigenome in GCA and this is a rich area for research. Epigenetics could underpin the variable clinical course of the disease.

## 13. Conclusions

It is now clear that in all the autoimmune rheumatic diseases there are various epigenetic aberrations (Figure 1). Each specific disease is likely to have its own epigenetic signature; for example, RA appears to be hypomethylated whereas in SSc the fibroblasts, at least, appear hypermethylated. Thus, different approaches to treatment will be warranted. It is likely that in the hypermethylated state in SSc the use of decitabine may be useful but in RA where the fibroblasts are already hypermethylated this would exacerbate the situation. Histone modifications are also likely to differ in different diseases and any drugs that target specific histone modifications must be used with knowledge of the precise histone modifications occurring in that particular setting. This will be critical in the treatment regime. Noncoding RNAs like miRNAs are now emerging as excellent druggable targets; however, issues regarding their stability and targeting* in vivo* still remain unclear. How do we get the right miR-mimic or antagomiR to the precise tissue? This is an active area of research and it is already bearing fruit with the use of aptamers. Epigenetic therapies are now coming to the fore and the use of the first miRNA therapy in HCV appears to be a success.

## Figures and Tables

**Table 1:** FDA-approved epigenetic drugs.

Drug	Epigenetic effect	Clinical trial
Miravirsen	Neutralisation of miRNA-122	Phase II [13]
MRX34	Ectopic expression of miRNA-34	Phase I [9]
Azacitidine (Vidaza)	DNA methyltransferase inhibitor	Phase III [15]
Decitabine (Dacogen)	DNA methyltransferase inhibitor	Phase III [17]
Vorinostat (Zolinza)	Pan-HDAC inhibitor	Phase II [24]

## References

[1] Alarcon G. S., Williams G. V., Singer J. Z. (1991). Early undifferentiated connective tissue disease. I. Early clinical manifestation in a large cohort of patients with undifferentiated connective tissue diseases compared with cohorts of well established connective tissue disease. *The Journal of Rheumatology*.

[2] Arango Duque G., Descoteaux A. (2014). Macrophage cytokines: involvement in immunity and infectious diseases. *Frontiers in Immunology*.

[3] Lu Y., Wahl L. M. (2005). Oxidative stress augments the production of matrix metalloproteinase-1, cyclooxygenase-2, and prostaglandin E2 through enhancement of NF-*κ*B activity in lipopolysaccharide-activated human primary monocytes. *Journal of Immunology*.

[4] Aggarwal A. (2014). Role of autoantibody testing. *Best Practice & Research Clinical Rheumatology*.

[5] Qu N., Xu M., Mizoguchi I. (2013). Pivotal roles of T-helper 17-related cytokines, IL-17, IL-22, and IL-23, in inflammatory diseases. *Clinical and Developmental Immunology*.

[6] Feinberg A. P. (2007). Phenotypic plasticity and the epigenetics of human disease. *Nature*.

[7] Wahren-Herlenius M., Dörner T. (2013). Immunopathogenic mechanisms of systemic autoimmune disease. *The Lancet*.

[8] Gervin K., Vigeland M. D., Mattingsdal M. (2012). DNA methylation and gene expression changes in monozygotic twins discordant for psoriasis: identification of epigenetically dysregulated genes. *PLoS Genetics*.

[9] Adams B. D., Parsons C., Slack F. J. (2015). The tumor-suppressive and potential therapeutic functions of miR-34a in epithelial carcinomas. *Expert Opinion on Therapeutic Targets*.

[10] Lu J., Clark A. G. (2012). Impact of microRNA regulation on variation in human gene expression. *Genome Research*.

[11] Hua X.-M., Wang J., Qian D.-M. (2015). DNA methylation level of promoter region of activating transcription factor 5 in glioma. *Journal of Zhejiang University Science B*.

[12] Weber M., Hellmann I., Stadler M. B. (2007). Distribution, silencing potential and evolutionary impact of promoter DNA methylation in the human genome. *Nature Genetics*.

[13] Janssen H. L. A., Reesink H. W., Lawitz E. J. (2013). Treatment of HCV infection by targeting microRNA. *The New England Journal of Medicine*.

[14] Dolinoy D. C. (2008). The agouti mouse model: an epigenetic biosensor for nutritional and environmental alterations on the fetal epigenome. *Nutrition Reviews*.

[15] Raj K., Mufti G. J. (2006). Azacytidine (Vidaza^®^) in the treatment of myelodysplastic syndromes. *Therapeutics and Clinical Risk Management*.

[16] Wu D., Du X., Jin J. (2015). Decitabine for treatment of myelodysplastic syndromes in Chinese patients: an open-label, phase-3b study. *Advances in Therapy*.

[17] Lübbert M., Suciu S., Hagemeijer A. (2016). Decitabine improves progression-free survival in older high-risk MDS patients with multiple autosomal monosomies: results of a subgroup analysis of the randomized phase III study 06011 of the EORTC Leukemia Cooperative Group and German MDS Study Group. *Annals of Hematology*.

[18] Zhang H., Zhang X., Clark E., Mulcahey M., Huang S., Shi Y. G. (2010). TET1 is a DNA-binding protein that modulates DNA methylation and gene transcription via hydroxylation of 5-methylcytosine. *Cell Research*.

[19] de Andres M. C., Perez-Pampin E., Calaza M. (2015). Assessment of global DNA methylation in peripheral blood cell subpopulations of early rheumatoid arthritis before and after methotrexate. *Arthritis Research and Therapy*.

[20] Rohle D., Popovici-Muller J., Palaskas N. (2013). An inhibitor of mutant IDH1 delays growth and promotes differentiation of glioma cells. *Science*.

[21] Chaturvedi A., Araujo Cruz M. M., Jyotsana N. (2013). Mutant IDH1 promotes leukemogenesis in vivo and can be specifically targeted in human AML. *Blood*.

[22] Mersfelder E. L., Parthun M. R. (2006). The tale beyond the tail: histone core domain modifications and the regulation of chromatin structure. *Nucleic Acids Research*.

[23] Bannister A. J., Kouzarides T. (2011). Regulation of chromatin by histone modifications. *Cell Research*.

[24] Wozniak M. B., Villuendas R., Bischoff J. R. (2010). Vorinostat interferes with the signaling transduction pathway of T-cell receptor and synergizes with phosphoinositide-3 kinase inhibitors in cutaneous T-cell lymphoma. *Haematologica*.

[25] *Horizon 2020 Framework Programme EULAR's position and recommendations*. http://www.eular.org/myUploadData/files/EU_Horizon_2020_EULAR_position_paper.pdf.

[26] Humby F., Bombardieri M., Manzo A. (2009). Ectopic lymphoid structures support ongoing production of class-switched autoantibodies in rheumatoid synovium. *PLoS Medicine*.

[27] Niimoto T., Nakasa T., Ishikawa M. (2010). MicroRNA-146a expresses in interleukin-17 producing T cells in rheumatoid arthritis patients. *BMC Musculoskeletal Disorders*.

[28] Müller N., Döring F., Klapper M. (2014). Interleukin-6 and Tumour Necrosis Factor-*α* differentially regulate lincRNA transcripts in cells of the innate immune system in vivo in human subjects with rheumatoid arthritis. *Cytokine*.

[29] Song J., Kim D., Han J., Kim Y., Lee M., Jin E.-J. (2014). PBMC and exosome-derived Hotair is a critical regulator and potent marker for rheumatoid arthritis. *Clinical and Experimental Medicine*.

[30] Liu C.-C., Fang T.-J., Ou T.-T. (2011). Global DNA methylation, DNMT1, and MBD2 in patients with rheumatoid arthritis. *Immunology Letters*.

[31] Karouzakis E., Rengel Y., Jüngel A. (2011). DNA methylation regulates the expression of CXCL12 in rheumatoid arthritis synovial fibroblasts. *Genes and Immunity*.

[32] Ishida K., Kobayashi T., Ito S. (2012). Interleukin-6 gene promoter methylation in rheumatoid arthritis and chronic periodontitis. *Journal of Periodontology*.

[33] Glossop J. R., Emes R. D., Nixon N. B. (2014). Genome-wide DNA methylation profiling in rheumatoid arthritis identifies disease-associated methylation changes that are distinct to individual T- and B-lymphocyte populations. *Epigenetics*.

[34] Altorok N., Coit P., Hughes T. (2014). Genome-wide DNA methylation patterns in naive CD4^+^ t cells from patients with primary Sjögren's syndrome. *Arthritis and Rheumatology*.

[35] O'Reilly S., Cant R., Ciechomska M., van Laar J. M. (2013). Interleukin-6: a new therapeutic target in systemic sclerosis?. *Clinical & Translational Immunology*.

[36] Fu L.-H., Ma C.-L., Cong B., Li S.-J., Chen H.-Y., Zhang J.-G. (2011). Hypomethylation of proximal CpG motif of interleukin-10 promoter regulates its expression in human rheumatoid arthritis. *Acta Pharmacologica Sinica*.

[37] Huber L. C., Brock M., Hemmatazad H. (2007). Histone deacetylase/acetylase activity in total synovial tissue derived from rheumatoid arthritis and osteoarthritis patients. *Arthritis and Rheumatism*.

[38] Wada T. T., Araki Y., Sato K. (2014). Aberrant histone acetylation contributes to elevated interleukin-6 production in rheumatoid arthritis synovial fibroblasts. *Biochemical and Biophysical Research Communications*.

[39] York M. R., Nagai T., Mangini A. J., Lemaire R., Van Seventer J. M., Lafyatis R. (2007). A macrophage marker, siglec-1, is increased on circulating monocytes in patients with systemic sclerosis and induced by type I interferons and toll-like receptor agonists. *Arthritis and Rheumatism*.

[40] Alsaleh G., François A., Philippe L. (2014). MiR-30a-3p negatively regulates BAFF synthesis in systemic sclerosis and rheumatoid arthritis fibroblasts. *PLoS ONE*.

[41] Wang Y., Shu Y., Xiao Y. (2014). Hypomethylation and overexpression of ITGAL (CD11a) in CD4^+^ T cells in systemic sclerosis. *Clinical Epigenetics*.

[42] Ciechomska M., Huigens C. A., Hügle T. (2013). Toll-like receptor-mediated, enhanced production of profibrotic TIMP-1 in monocytes from patients with systemic sclerosis: role of serum factors. *Annals of the Rheumatic Diseases*.

[43] O'Reilly S., Cant R., Ciechomska M. (2014). Serum amyloid A induces interleukin-6 in dermal fibroblasts via Toll-like receptor 2, interleukin-1 receptor-associated kinase 4 and nuclear factor-*κ*B. *Immunology*.

[44] Ciechomska M., O'Reilly S., Przyborski S., Oakley F., Bogunia-Kubik K., van Laar J. M. (2016). Histone demethylation and toll-like receptor 8-dependent cross-talk in monocytes promotes transdifferentiation of fibroblasts in systemic sclerosis via fra-2. *Arthritis & Rheumatology*.

[45] Wang Y., Yang Y., Luo Y. (2013). Aberrant histone modification in peripheral blood B cells from patients with systemic sclerosis. *Clinical Immunology*.

[46] Primo M. N., Bak R. O., Schibler B., Mikkelsen J. G. (2012). Regulation of pro-inflammatory cytokines TNF*α* and IL24 by microRNA-203 in primary keratinocytes. *Cytokine*.

[47] Xia P., Fang X., Zhang Z.-H. (2012). Dysregulation of miRNA146a versus IRAK1 induces IL-17 persistence in the psoriatic skin lesions. *Immunology Letters*.

[48] Gu X., Nylander E., Coates P. J., Fahraeus R., Nylander K. (2015). Correlation between reversal of DNA methylation and clinical symptoms in psoriatic epidermis following narrow-band UVB phototherapy. *Journal of Investigative Dermatology*.

[49] Chen M., Chen Z.-Q., Cui P.-G. (2008). The methylation pattern of p16INK4a gene promoter in psoriatic epidermis and its clinical significance. *British Journal of Dermatology*.

[50] Tovar-Castillo L. E., Cancino-Díaz J. C., García-Vázquez F. (2007). Under-expression of VHL and over-expression of HDAC-1, HIF-1*α*, LL-37, and IAP-2 in affected skin biopsies of patients with psoriasis. *International Journal of Dermatology*.

[51] Zhang P., Su Y., Zhao M., Huang W., Lu Q. (2011). Abnormal histone modifications in PBMCs from patients with psoriasis vulgaris. *European Journal of Dermatology*.

[52] Quddus J., Johnson K. J., Gavalchin J. (1993). Treating activated CD4+ T cells with either of two distinct DNA methyltransferase inhibitors, 5-azacytidine or procainamide, is sufficient to cause a lupus-like disease in syngeneic mice. *Journal of Clinical Investigation*.

[53] Deng C., Kaplan M. J., Yang J. (2001). Decreased ras-mitogen-activated protein kinase signaling may cause DNA hypomethylation in T lymphocytes from lupus patients. *Arthritis and Rheumatism*.

[54] Li Y., Zhao M., Yin H. (2010). Overexpression of the growth arrest and DNA damage-induced 45*α* gene contributes to autoimmunity by promoting DNA demethylation in lupus T cells. *Arthritis and Rheumatism*.

[55] Li Y., Gorelik G., Strickland F. M., Richardson B. C. (2014). Oxidative stress, T cell dna methylation, and lupus. *Arthritis and Rheumatology*.

[56] Strickland F. M., Li Y., Johnson K., Sun Z., Richardson B. C. (2015). CD4^+^ T cells epigenetically modified by oxidative stress cause lupus-like autoimmunity in mice. *Journal of Autoimmunity*.

[57] Lu Q., Kaplan M., Ray D. (2002). Demethylation of ITGAL (CD11a) regulatory sequences in systemic lupus erythematosus. *Arthritis and Rheumatism*.

[58] Lu Q., Wu A., Tesmer L., Ray D., Yousif N., Richardson B. (2007). Demethylation of CD40LG on the inactive X in T cells from women with lupus. *The Journal of Immunology*.

[59] Coit P., Jeffries M., Altorok N. (2013). Genome-wide DNA methylation study suggests epigenetic accessibility andtranscriptional poising of interferon-regulated genes in naïve CD4+ T cellsfrom lupus patients. *Journal of Autoimmunity*.

[60] Absher D. M., Li X., Waite L. L. (2013). Genome-wide DNA methylation analysis of systemic lupus erythematosus reveals persistent hypomethylation of interferon genes and compositional changes to CD4+ T-cell populations. *PLoS Genetics*.

[61] Chung S. A., Nititham J., Elboudwarej E. (2015). Genome-wide assessment of differential DNA methylation associated with autoantibody production in systemic lupus erythematosus. *PLoS ONE*.

[62] Fali T., Le Dantec C., Thabet Y. (2014). DNA methylation modulates HRES1/p28 expression in B cells from patients with lupus. *Autoimmunity*.

[63] Qin H., Zhu X., Liang J. (2013). MicroRNA-29b contributes to DNA hypomethylation of CD4^+^ T cells in systemic lupus erythematosus by indirectly targeting DNA methyltransferase 1. *Journal of Dermatological Science*.

[64] Tang Y., Luo X., Cui H. (2009). MicroRNA-146a contributes to abnormal activation of the type I interferon pathway in human lupus by targeting the key signaling proteins. *Arthritis and Rheumatism*.

[65] Hong Y., Wu J., Zhao J. (2013). miR-29b and miR-29c are involved in toll-like receptor control of glucocorticoid-induced apoptosis in human plasmacytoid dendritic cells. *PLoS ONE*.

[66] Pan W., Zhu S., Yuan M. (2010). MicroRNA-21 and microRNA-148a contribute to DNA hypomethylation in lupus CD4+ T cells by directly and indirectly targeting DNA methyltransferase 1. *The Journal of Immunology*.

[67] Deng Y., Zhao J., Sakurai D. (2013). MicroRNA-3148 modulates allelic expression of toll-like receptor 7 variant associated with systemic lupus erythematosus. *PLoS Genetics*.

[68] Fan W., Liang D., Tang Y. (2012). Identification of microRNA-31 as a novel regulator contributing to impaired interleukin-2 production in T cells from patients with systemic lupus erythematosus. *Arthritis & Rheumatism*.

[69] Wang G., Tam L.-S., Kwan B. C.-H. (2012). Expression of miR-146a and miR-155 in the urinary sediment of systemic lupus erythematosus. *Clinical Rheumatology*.

[70] Hu N., Qiu X., Luo Y. (2008). Abnormal histone modification patterns in lupus CD4^+^ T cells. *The Journal of Rheumatology*.

[71] Mandal M., Powers S. E., Maienschein-Cline M. (2011). Epigenetic repression of the Igk locus by STAT5-mediated recruitment of the histone methyltransferase Ezh2. *Nature Immunology*.

[72] Long H., Huang W., Yin H., Zhao S., Zhao M., Lu Q. (2009). Abnormal expression pattern of histone demethylases in CD4 + T cells of MRL/lpr lupus-like mice. *Lupus*.

[73] Zhang Q., long H., Liao J. (2011). Inhibited expression of hematopoietic progenitor kinase 1 associated with loss of jumonji domain containing 3 promoter binding contributes to autoimmunity in systemic lupus erythematosus. *Journal of Autoimmunity*.

[74] Mishra N., Reilly C. M., Brown D. R., Ruiz P., Gilkeson G. S. (2003). Histone deacetylase inhibitors modulate renal disease in the MRL-lpr/lpr mouse. *Journal of Clinical Investigation*.

[75] Zhou Y., Qiu X., Luo Y. (2011). Histone modifications and methyl-CpG-binding domain protein levels at the TNFSF7 (CD70) promoter in SLE CD4+ T cells. *Lupus*.

[76] Pauley K. M., Stewart C. M., Gauna A. E. (2011). Altered miR-146a expression in Sjögren's syndrome and its functional role in innate immunity. *European Journal of Immunology*.

[77] Shi H., Zheng L.-Y., Zhang P., Yu C.-Q. (2014). miR-146a and miR-155 expression in PBMCs from patients with Sjögren's syndrome. *Journal of Oral Pathology and Medicine*.

[78] Zilahi E., Tarr T., Papp G., Griger Z., Sipka S., Zeher M. (2012). Increased microRNA-146a/b, TRAF6 gene and decreased IRAK1 gene expressions in the peripheral mononuclear cells of patients with Sjögren's syndrome. *Immunology Letters*.

[79] Tandon M., Gallo A., Jang S.-I., Illei G. G., Alevizos I. (2012). Deep sequencing of short RNAs reveals novel microRNAs in minor salivary glands of patients with Sjögren's syndrome. *Oral Diseases*.

[80] Liang C., Xiong K., Szulwach K. E. (2013). Sjögren syndrome antigen B (SSB)/La promotes global microRNA expression by binding microRNA precursors through stem-loop recognition. *Journal of Biological Chemistry*.

[81] Thabet Y., Le Dantec C., Ghedira I. (2013). Epigenetic dysregulation in salivary glands from patients with primary Sjögren's syndrome may be ascribed to infiltrating B cells. *Journal of Autoimmunity*.

[82] Yu X., Liang G., Yin H. (2013). DNA hypermethylation leads to lower FOXP3 expression in CD4+ T cells of patients with primary Sjögren's syndrome. *Clinical Immunology*.

[83] Altorok N., Coit P., Hughes T. (2014). Genome-wide DNA methylation patterns in naive cd4+ t cells from patients with primary sjögren's syndrome. *Arthritis and Rheumatology*.

[84] Lai N.-S., Yu H.-C., Chen H.-C., Yu C.-L., Huang H.-B., Lu M.-C. (2013). Aberrant expression of microRNAs in T cells from patients with ankylosing spondylitis contributes to the immunopathogenesis. *Clinical & Experimental Immunology*.

[85] Qi J., Hou S., Zhang Q. (2013). A functional variant of pre-miRNA-196a2 confers risk for Behcet's disease but not for Vogt-Koyanagi-Harada syndrome or AAU in ankylosing spondylitis. *Human Genetics*.

[86] Xia Y., Chen K., Zhang M.-H. (2015). MicroRNA-124 involves in ankylosing spondylitis by targeting ANTXR2. *Modern Rheumatology*.

[87] Niu Z., Wang J., Zou H., Yang C., Huang W., Jin L. (2015). Common MIR146A polymorphisms in Chinese ankylosing spondylitis subjects and controls. *PLoS ONE*.

[88] Appel H., Wu P., Scheer R. (2011). Synovial and peripheral blood CD4+FoxP3+ T cells in spondyloarthritis. *The Journal of Rheumatology*.

[89] Lai N.-S., Chou J.-L., Chen G. C. W., Liu S.-Q., Lu M.-C., Chan M. W. Y. (2014). Association between cytokines and methylation of SOCS-1 in serum of patients with ankylosing spondylitis. *Molecular Biology Reports*.

[90] Toussirot E., Abbas W., Khan K. A. (2013). Imbalance between HAT and HDAC activities in the PBMCs of patients with ankylosing spondylitis or rheumatoid arthritis and influence of HDAC inhibitors on TNF alpha production. *PLoS ONE*.

[91] Toussirot É., Wendling D., Herbein G. (2014). Biological treatments given in patients with rheumatoid arthritis or ankylosing spondylitis modify HAT/HDAC (histone acetyltransferase/histone deacetylase) balance. *Joint Bone Spine*.

[92] Roberts A. R., Vecellio M., Chen L. (2016). An ankylosing spondylitis-associated genetic variant in the *IL23R-IL12RB2* intergenic region modulates enhancer activity and is associated with increased Th1-cell differentiation. *Annals of the Rheumatic Diseases*.

[93] Coit P., De Lott L. B., Nan B., Elner V. M., Sawalha A. H. (2016). DNA methylation analysis of the temporal artery microenvironment in giant cell arteritis. *Annals of the Rheumatic Diseases*.

[94] Croci S., Zerbini A., Boiardi L. (2016). MicroRNA markers of inflammation and remodelling in temporal arteries from patients with giant cell arteritis. *Annals of the Rheumatic Diseases*.

